# Endogenous retrovirus group FRD member 1 is a potential biomarker for prognosis and immunotherapy for kidney renal clear cell carcinoma

**DOI:** 10.3389/fcimb.2023.1252905

**Published:** 2023-09-13

**Authors:** Xiaofen Wen, Jiaxin Shen, Maria Rosaria De Miglio, De Zeng, Leonardo A. Sechi

**Affiliations:** ^1^ Department of Biomedical Sciences, University of Sassari, Sassari, Italy; ^2^ Department of Medical Oncology, Cancer Hospital of Shantou University Medical College, Shantou, Guangdong, China; ^3^ Department of Hematology, The First Affiliated Hospital of Shantou University Medical College, Shantou, Guangdong, China; ^4^ Department of Medical, Surgical and Experimental Sciences, University of Sassari, Sassari, Italy; ^5^ Struttura Complessa (SC) Microbiologia e Virologia, Azienda Ospedaliera Universitaria, Sassari, Italy

**Keywords:** endogenous retrovirus, biomarker, immunotherapy, kidney renal clear cell carcinoma, bioinformatics

## Abstract

**Introduction:**

The activation of endogenous retroviral (ERV) genes in kidney renal clear cell carcinoma (KIRC) suggests the necessity for further research on their functions.

**Methods:**

In this study, KIRC and healthy cohorts were obtained from TGGA and GEO datasets. Subsequently, differential analysis and functional annotation were conducted using GO, KEGG, and GSEA. Clinical outcomes were then observed and utilized in the development of a nomogram.

**Results:**

We observed the general low expression of ERVFRD-1 in KIRC tumors compared to normal tissue (P < 0.001) across multiple cohorts. Differential analysis and functional annotation using GO, KEGG, GSEA analysis revealed significant involvement of ERVFRD-1 in tumor immunoregulation: a close relation to the infiltration levels of mast cells and Treg cell (P < 0.001) and occurrence with a variety of immune markers. Methylation status was then applied to uncover potential mechanisms of ERVFRD-1 in KIRC. Notably, higher expression levels of ERVFRD-1 were associated with extended overall survival, disease-specific survival, and progression-free survival. Finally, based on Cox regression analysis, we constructed a nomogram incorporating ERVFRD-1, pathologic T, and age, which exhibited promising predictive power in assessing the survival outcomes of KIRC patients.

**Discussion:**

To sum up, our study suggests that ERVFRD-1 plays a role in regulating immunological activity within the tumor microenvironment and is associated with overall survival in KIRC patients. ERVFRD-1 may therefore be a sensitive biomarker for diagnosis, immunotherapy, and prognosis assessment of KIRC.

## 1 Introduction

According to the latest GLOBOCAN report, kidney cancer is ranked 16th among the 36 types of cancers worldwide, with an estimation of over 431,000 new cases (accounting for 2.2% of all cancer types) and approximately 179,000 deaths (1.8% of all cancer-related deaths) (Sung et al., 2021). Kidney cancer is typically divided into three histological types: clear cell (TCGA-KIRC, WHO-ccRCC), papillary (TCGA-KIRP, WHO-ChRCC), and chromophobe (TCGA-KICH, WHO-PRCC) (Moch et al., 2022). KIRC is the most prevalent type, representing over 80% of all kidney cancer cases. Unfortunately, KIRC is highly malignant and not responsive to most chemotherapy or radiotherapy (Makhov et al., 2018). While surgical resection is currently an effective treatment for early-stage localized KIRC (Porta et al., 2019), around 25% to 50% of primary patients experience recurrence within five years following nephrectomy, and one-third of patients develop metastases (Kotecha et al., 2019). Therefore, it is urgent to discover novel and valuable biomarkers for early diagnosis and treatment of this particular type of kidney cancer.

Human endogenous retroviruses (HERVs) are estimated to make up approximately 8% of the human genome (Geis and Goff, 2020). These retroviruses are typically inherited through the germ line. Due to multiple mutations in their genomes, HERVs are usually defective and rarely capable of producing viral particles. Despite this, HERVs-derived proteins can significantly influence the regulation of gene expression and cellular functions in the host. Apart from viral transcripts, ERV-encoded proteins translated from intact open reading frames (ORFs) have been implicated in both pathological and physiological processes (Turner et al., 2001). Numerous studies have demonstrated that HERV families are transcribed in human tissues during various stages of development, in both healthy and diseased conditions (Suntsova et al., 2015). HERVs have therefore been associated with several biological processes, including placentation, stemness, aging, immune responses, neurodegeneration, autoimmunity, and carcinogenesis (Okahara et al., 2004; Balada et al., 2009; Zhang et al., 2019; Nair et al., 2021; Rangel et al., 2022).

One particular HERV, ERVFRD-1, produces a protein called Syncytin-2, which plays a significant role during embryogenesis by contributing to placental formation and facilitating immune tolerance of the maternal system towards the developing fetus. In an *in vitro* experiment, it was observed that MCA205 cells transfected with syncytin-2 exhibited long-lasting tumorigenic potential when engrafted into mice (Mangeney et al., 2007). Moreover, increased expression of ERVFRD-1 in cancers from the Cancer Genome Atlas (TCGA) with ERVFRD-1 fusions suggested that ERVFRD-1 promoted tumor growth by inhibiting the anti-tumor immune response of the host mice. This finding suggests that enhanced expression of ERVFRD-1 in human cancer cells could potentially contribute to tumor growth as well (Namba et al., 2021). However, the precise role of ERVFRD-1 in KIRC has not been investigated.

Cancer immunotherapy has emerged as a potential alternative or complement to traditional cytotoxic chemotherapy and radiotherapy. However, the effectiveness of immune checkpoint blockades (ICBs), a key approach in immunotherapy, is significantly influenced by the tumor microenvironment (Li et al., 2021). KIRC patients often exhibit higher immune-related scores. Interestingly, studies have linked HERVs expression to response to ICBs in certain cancer types. In both human and mouse lung adenocarcinomas, researchers have discovered that tumor-binding antibodies and local germinal center responses are elicited, with ERV-envelope glycoproteins identified as the primary anti-tumor antibody target. Additionally, ICBs are capable of amplifying the B cell responses within this process by targeting HERVs in both humans and mice (Ng et al., 2023). A different HERV pattern of association with the outcome of KIRC PD-L1 immunotherapy was observed by Lewis Au et al (Au et al., 2021). However, despite the known role of the ERVFRD-1 protein in suppressing immunity during placental development, its immunological impact in KIRC and its potential relationship with the effectiveness of immunotherapy remain unexplored. Therefore, further research is necessary to clarify the relationship between ERVFRD-1 and immunity in KIRC.

The objective of this study is to utilize bioinformatics to examine ERVFRD-1 expression and its clinicopathological and prognostic significance, as well as the underlying molecular mechanisms and immune cell infiltration in KIRC. By investigating these aspects, our study seeks to provide valuable insights that could assist clinicians in refining treatment strategies and enhancing outcomes for patients diagnosed with KIRC.

## 2 Materials and methods

### 2.1 Data collection and processing

We collected mRNA expression profiles and corresponding clinical characteristics of KIRC patients from two databases: the Cancer Genome Atlas (TCGA, https://portal.gdc.cancer.gov) database (n = 532), and the Gene Expression Omnibus database (GEO, http://www.ncbi.nlm.nih.gov/geo/) (n = 144). The data obtained from these databases were in level 3 HTSeq-FPKM format and were normalized as transcripts per million reads (TPM). For a broader analysis encompassing multiple cancer types, we also retrieved RNA-sequencing data in TPM format from the University of California, Santa Cruz Xena (UCSC Xena website, https://xenabrowser.net/datapages/). The RNA-seq data of 33 pan-cancer samples including adrenocortical carcinoma (ACC), bladder cancer (BLCA), breast carcinoma (BRCA), cervical cancer (CESC), cholangiocarcinoma (CHOL), colorectal cancer (COAD), diffused large B-cell lymphoma (DLBC), esophageal cancer (ESCA), glioblastoma (GBM), head and neck squamous cell carcinoma (HNSC), kidney chromophobe (KICH), kidney renal clear cell carcinoma (KIRC), kidney renal papillary cell carcinoma (KIRP), Acute Myeloid Leukemia (LAML), low-grade glioma (LGG), liver hepatocellular carcinoma(LIHC), lung adenocarcinoma (LUAD), lung squamous cell carcinoma (LUSC), mesothelioma (MESO), ovarian serous cystadenocarcinoma (OV), pancreatic adenocarcinoma (PAAD), pheochromocytoma and paraganglioma (PCPG), prostate cancer (PRAD), rectal cancer (READ), sarcoma (SARC), skin cutaneous melanoma (SKCM), stomach adenocarcinoma (STAD), testicular cancer (TGCT), thyroid carcinoma (THCA), thymoma (THYM), uterine corpus endometrial carcinoma (UCEC), uterine carcinosarcoma (UCS) and ocular melanomas (UVM), were obtained from TCGA. Normal samples were selected from the Genotype-Tissue Expression (http://commonfund.nih.gov/GTEx/) dataset.

### 2.2 Analysis of Differentially Expressed Genes (DEGs)

The cohort of patients with KIRC from TCGA was categorized into high and low ERVFRD-1 expression groups using the median ERVFRD-1 expression score. DEG analysis was performed between these two groups using the R package DESeq2 (Robinson et al., 2010; Love et al., 2014). The significance threshold was set at an adjusted P value of < 0.05, and a |log2-fold-change (FC)| > 1. We used Spearman’s correlation analysis to evaluate the association between ERVFRD-1 expression and the top 10 DEGs.

### 2.3 Protein-Protein Interaction (PPI) network analysis

Following the differential gene expression analysis, we constructed a PPI network using the Retrieval of Interacting Genes online database (STRING, https://www.string-db.org/). A confidence score threshold of > 0.7 was set, while other parameters were kept at their default values. The resulting PPI network was then visualized using Cytoscape software (version 3.9.1) (Lotia et al., 2013). Next, we used CytoHubba, a plugin within Cytoscape software, to identify the top 10 hub genes among these DEGs (Chin et al., 2014).

### 2.4 Functional enrichment analysis

To gain insights into the functional implications of the DEGs, we performed functional enrichment analyses, specifically Gene Ontology (GO) and Kyoto Encyclopedia of Genes and Genomes (KEGG) analyses. These analyses were conducted using the R package ggplot2 (version 3.3.6). Additionally, we employed gene set enrichment analysis (GSEA) with the R package clusterProfiler (version 4.2.1) (Subramanian et al., 2005; Yu et al., 2012). We considered function or pathway terms with an adjusted P value < 0.05 and false discovery rate (FDR) < 0.25 as statistically significant enrichment results.

### 2.5 Immune infiltration analysis

To assess the extent of immune infiltration in KIRC, we utilized the “estimate” package in R to calculate the stromal, immune scores and ESTIMATE scores (Yoshihara et al., 2013), and conduct a comprehensive analysis involving 24 immune cell types, visualized by ggplot2[3.3.6]. The single-sample Gene Set Enrichment Analysis (ssGSEA) technique was then employed to evaluate the relative enrichment score of these immune cells. This analysis was performed using the GSVA R package (Bindea et al., 2013; Hänzelmann et al., 2013). Furthermore, we examined the correlation between the expression of ERVFRD-1 and these immune cells using Spearman’s correlation analysis. The Wilcoxon rank sum test was utilized to determine differences in immune infiltration levels between groups with high and low ERVFRD-1 expression. Additionally, to examine the interaction between tumor immunity and markers, we utilized the ggstatsplot R package to analyze the correlation between the enrichment score of tumor mutation burden (TMB) and ERVFRD-1 expression. Then potential ICB response was predicted using the TIDE algorithm (Jiang et al., 2018), visualized by ggplot2(v3.3.3) and ggpubr(0.4.0).

### 2.6 DNA methylation analysis

To explore the underlying mechanism of ERVFRD-1 in KIRC, we employed the MethSurv database (https://biit.cs.ut.ee/methsurv/) to evaluate the DNA methylation status of ERVFRD-1 and prognostic value of ERVFRD-1 methylation levels. MethSurv is an online tool that allows for multivariable survival analysis based on DNA methylation data (Modhukur et al., 2018).

### 2.7 Survival analysis

We performed survival analysis using the Kaplan-Meier method and assessed statistical significance with the log-rank test, where the median expression level of ERVFRD-1 was used as the cut-off value. To evaluate the impact of clinical variables on patient outcomes, we conducted univariate and multivariate Cox regression analyses. In the univariate Cox regression analysis, prognostic variables with a significance level of P < 0.1 were included in the multivariate Cox regression analysis. The results were visualized in a forest plot using the R package ggplot2.

### 2.8 Construction and validation of the nomogram

We created a nomogram using the results from multivariate Cox analysis that included independent prognostic factors such as clinical characteristics and risk scores of KIRC patients. This nomogram aimed to predict the overall survival probability over 1-, 3-, and 5-year periods. The performance of the nomogram was evaluated using calibration plots while the concordance index (C-index) was utilized to quantify its discriminatory ability. The R package RMS (version 6.2-0) was utilized to create the nomogram and calibration plots. Additionally, we evaluated the predictive accuracy of the model using time-dependent receiver-operating characteristic (ROC) curves generated through the time ROC package.

### 2.9 Statistical analysis

R software (version 3.6.3) was used to conduct all statistical analyses. We evaluated the statistical significance of ERVFRD-1 expression in non-paired and paired tissues using the Wilcoxon rank-sum test and paired-sample t-test, respectively. The correlation between clinical features and ERVFRD-1 expression was assessed using both the Wilcoxon rank-sum test and logistic regression. All tests conducted were two-sided, and we regarded P values < 0.05 as statistically significant.

## 3 Results

### 3.1 Patient characteristics

We collected clinical and RNA-sequencing data for 532 patients from TCGA datasets, which consisted of 72 patients with matched normal tissue samples. The **Supplementary Table S1** shows the clinicopathological characteristics of these patients. To augment the sample size, we acquired gene expression data from normal kidney tissues (n = 28) in the GTEx datasets. Furthermore, another dataset from the GEO dataset GSE53757 was introduced, which contained normal kidney tissues (n=71) and KIRC tissues (n=73).

### 3.2 Low expression of ERVFRD-1 associated with prognosis value in KIRC

**Figure 1:**
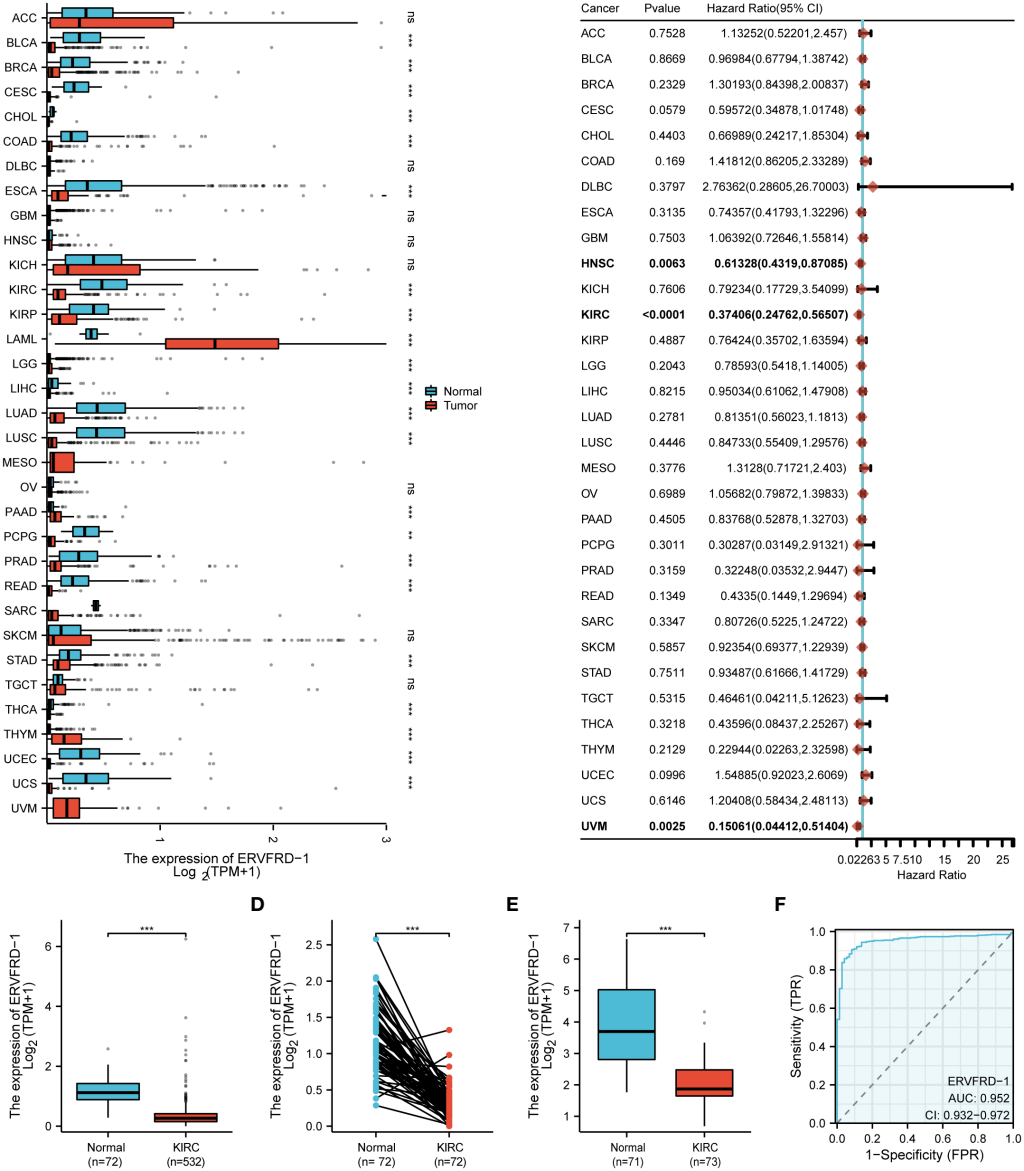
The expression level of ERVFRD-1 in pan-cancer including KIRC. **(A)** Expression of ERVFRD-1 in different types of tumors compared with normal tissues in TCGA and GTEx databases; **(B)** Overall survival of ERVFRD-1 in different types of tumors in TCGA; **(C, D)** in non-matched and matched normal kidney tissues vs KIRC from TCGA; **(E)** in normal vs KIRC tissues from datasets GSE53757. **(F)** ROC curve to classify KIRC vs normal kidney tissues in the TCGA database. TCGA, The Cancer Genome Atlas; GTEx, Genotype Tissue Expression Project; GEO, Gene Expression Omnibus. **P < 0.01, ***P < 0.001. ns, non significant.

Based on the pan-cancer analysis, ERVFRD-1 expression was generally low in various types of cancers such as BLCA, BRCA, CESC, CHOL, COAD, ESCA, KIRC, KIRP, LGG, LIHC, LUSC, PCPG, PRAD, READ, STAD, THCA, UCEC, and UCS (**Figure 1A**). Additionally, ERVFRD-1 expression was found to be significantly associated with prognosis in KIRC (**Figure 1B**). To further investigate the role of ERVFRD-1 in KIRC, we analyzed its expression in TCGA and GEO databases. As illustrated in **Figure 1C**, ERVFRD-1 expression was markedly lower in KIRC than in normal kidney tissues (p < 0.001). This trend was also observed in 72 paired kidney tissues (p < 0.001) (**Figure 1D**). Similarly, a significant decrease in ERVFRD-1 expression was found in KIRC compared to normal tissues in the GEO dataset GSE53757 (**Figure 1E**). Moreover, ERVFRD-1 expression demonstrated better predictive ability in distinguishing KIRC from normal tissues, exhibiting an area under the curve (AUC) of 0.952 (95% confidence interval [CI]=0.932-0.972) as indicated by the ROC curve (**Figure 1F**).

### 3.3 Association of ERVFRD-1 expression levels with clinical characteristics of KIRC

**Figure 2:**
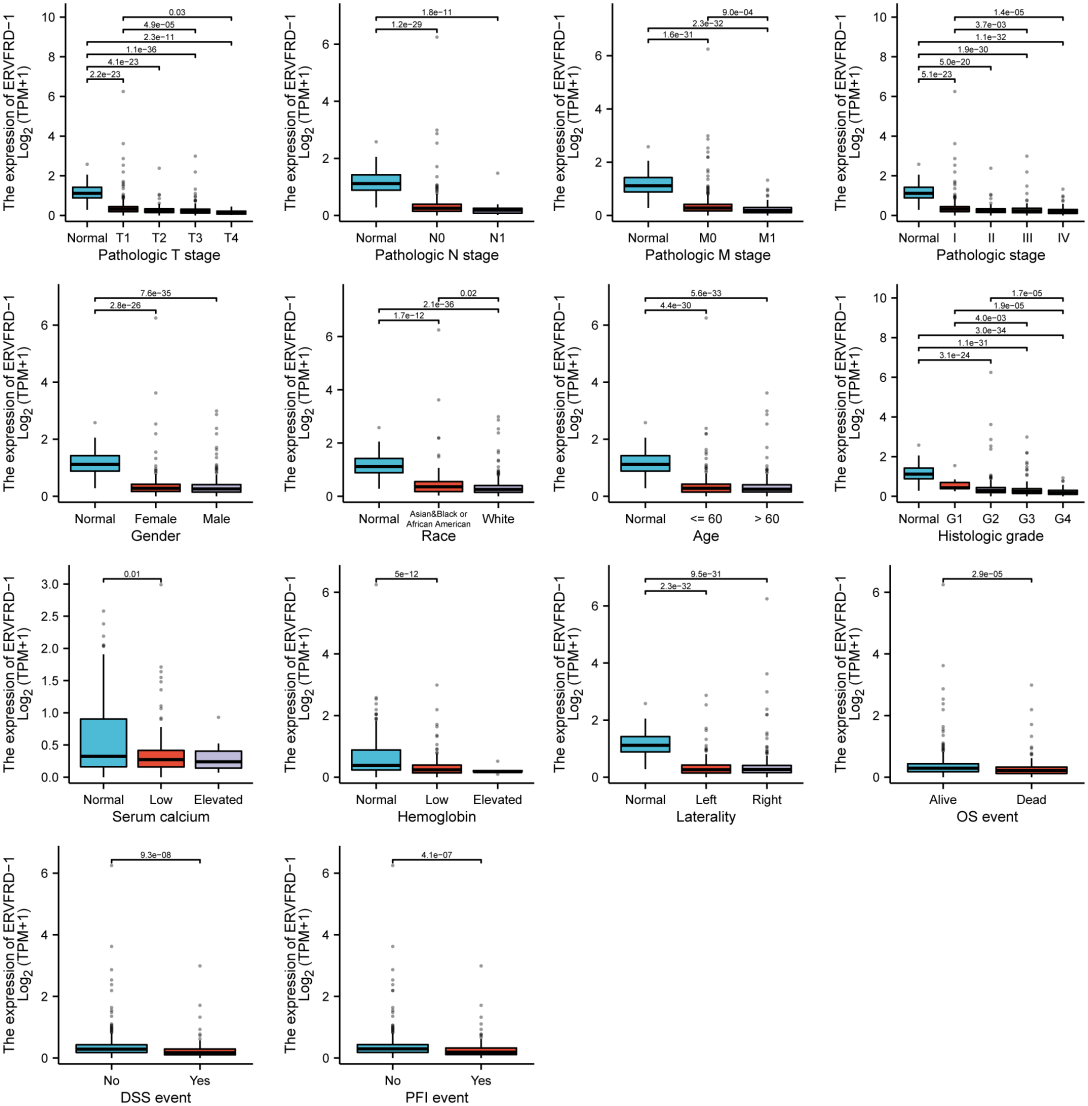
Associations between ERVFRD-1 expression and clinicopathological characteristics. Data are shown for the T stage, N stage, M stage, pathologic stage, gender, race, age, histologic grade, serum calcium, hemoglobin, laterality, OS event, DSS event, and PFI event. OS, overall survival; DSS, disease-specific survival; PFI, progression free interval.

We utilized the TCGA database to investigate the impact of various pathologic features on ERVFRD-1 transcription in KIRC samples (**Table 1**). As displayed in **Figure 2**, ERVFRD-1 expression remained consistently lower in KIRC patients than in controls across different sub-groups. The sub-group analysis, which included T stage, N stage, M stage, pathologic stage, gender, race, age, histology grade, serum_calcium, hemoglobin, laterality, OS event, DSS event, and PFI event, also indicated a significant reduction in ERVFRD-1 expression in KIRC patients compared to controls. Moreover, the univariate logistic regression analyses showed statistically significant differences between the high and low ERVFRD-1 expression groups in terms of T stage (T3 & T4 vs. T1 & T2, OR = 0.414, 95% CI = 0.288 - 0.596, P < 0.001), M stage (M1 vs. M0, OR = 0.411, 95% CI = 0.245 - 0.688, P < 0.001), pathologic stage (Stage III & IV vs. Stage I & II, OR = 0.389, 95% CI = 0.272 - 0.557, P < 0.001), gender (Male vs. Female, OR = 0.604, 95% CI = 0.422 - 0.864, P = 0.006), race (white vs. Asian & Black or African American, OR = 0.368, 95% CI = 0.209 - 0.647, P < 0.001), and histologic grade (G3 & G4 vs. G1 & G2, OR = 0.449, 95% CI = 0.317 - 0.635, P < 0.001), respectively (**Table 2**).

**Table 1:** Clinicopathological characteristics of high- and low-ERVFRD-1 expression groups (n = 532).

Characteristics	Low expression of ERVFRD-1	High expression of ERVFRD-1	pvalue
n	266	266	
Age, median (IQR)	62 (53.25, 71)	59 (51, 68)	0.007
Pathologic T stage, n (%)			2.35E-05
T1	109 (20.5%)	163 (30.6%)	
T2	40 (7.5%)	29 (5.5%)	
T3	108 (20.3%)	72 (13.5%)	
T4	9 (1.7%)	2 (0.4%)	
Pathologic N stage, n (%)			0.080432
N0	126 (49.2%)	114 (44.5%)	
N1	12 (4.7%)	4 (1.6%)	
Pathologic M stage, n (%)			0.000436
M0	197 (39.4%)	224 (44.8%)	
M1	54 (10.8%)	25 (5%)	
Pathologic stage, n (%)			1.43E-05
Stage I	105 (19.8%)	161 (30.4%)	
Stage II	33 (6.2%)	24 (4.5%)	
Stage III	69 (13%)	54 (10.2%)	
Stage IV	56 (10.6%)	27 (5.1%)	
Gender, n (%)			0.413772
Female	89 (16.7%)	98 (18.4%)	
Male	177 (33.3%)	168 (31.6%)	
Race, n (%)			0.031509
Asian&Black or African American	24 (4.6%)	40 (7.6%)	
White	239 (45.5%)	222 (42.3%)	
Age, n (%)			0.056431
<= 60	121 (22.7%)	143 (26.9%)	
> 60	145 (27.3%)	123 (23.1%)	
Histologic grade, n (%)			4.93E-06
G1	1 (0.2%)	13 (2.5%)	
G2	100 (19.1%)	128 (24.4%)	
G3	106 (20.2%)	100 (19.1%)	
G4	54 (10.3%)	22 (4.2%)	
Serum calcium, n (%)			0.08779
Low	100 (27.5%)	104 (28.6%)	
Normal	91 (25%)	59 (16.2%)	
Elevated	6 (1.6%)	4 (1.1%)	
Hemoglobin, n (%)			0.016122
Low	147 (32.5%)	115 (25.4%)	
Normal	81 (17.9%)	104 (23%)	
Elevated	4 (0.9%)	1 (0.2%)	
Laterality, n (%)			0.63072
Left	128 (24.1%)	122 (23%)	
Right	138 (26%)	143 (26.9%)	
OS event, n (%)			0.001
Alive	161 (30.3%)	196 (36.8%)	
Dead	105 (19.7%)	70 (13.2%)	
DSS event, n (%)			< 0.001
Alive	184 (35.3%)	228 (43.8%)	
Dead	77 (14.8%)	32 (6.1%)	
PFI event, n (%)			2.17E-05
No	163 (30.6%)	208 (39.1%)	
Yes	103 (19.4%)	58 (10.9%)	

**Table 2:** Associations of ERVFRD-1 expression with clinicopathological characteristics of KIRC patients (n = 532).

Characteristics	Total (N)	OR (95% CI)	P value
Pathologic T stage (T3&T4 vs. T1&T2)	541	0.414 (0.288 - 0.596)	< 0.001
Pathologic N stage (N1 vs. N0)	258	0.580 (0.205 - 1.647)	0.307
Pathologic M stage (M1 vs. M0)	508	0.411 (0.245 - 0.688)	< 0.001
Pathologic stage (Stage III&Stage IV vs. Stage I&Stage II)	538	0.389 (0.272 - 0.557)	< 0.001
Gender (Male vs. Female)	541	0.604 (0.422 - 0.864)	0.006
Race (White vs. Asian&Black or African American)	534	0.368 (0.209 - 0.647)	< 0.001
Age (> 60 vs. <= 60)	541	0.856 (0.611 - 1.200)	0.367
Histologic grade (G3&G4 vs. G1&G2)	533	0.449 (0.317 - 0.635)	< 0.001
Serum calcium (Elevated vs. Low)	214	0.455 (0.114 - 1.807)	0.263
Hemoglobin (Elevated vs. Low)	269	0.891 (0.146 - 5.419)	0.900
Laterality (Right vs. Left)	540	1.143 (0.815 - 1.603)	0.438

KIRC, kidney renal clear cell Carcinoma.

### 3.4 Identification of DEGs in KIRC and PPI network analysis

**Figure 3:**
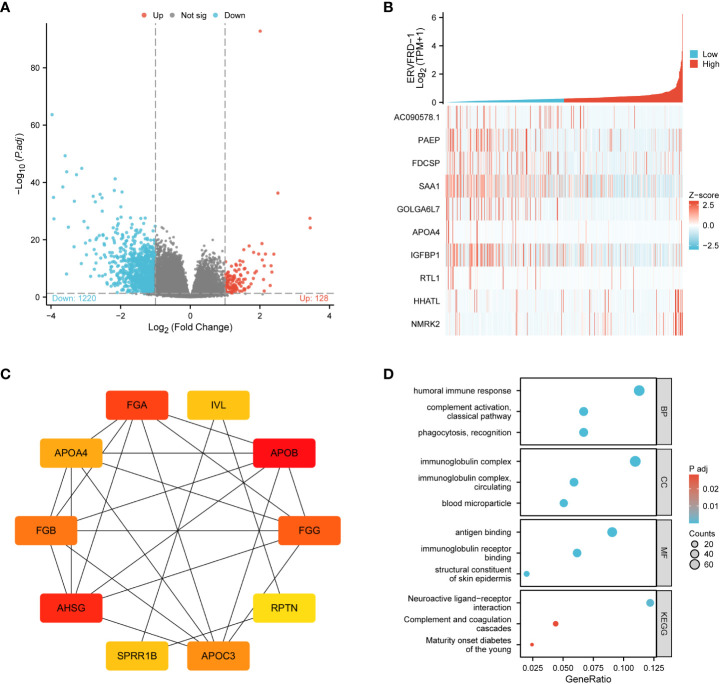
ERVFRD-1 related DEGs and functional enrichment analysis of ERVFRD-1 in KIRC using GO and KEGG. **(A)** Volcano plot of DEGs. Blue and red dots indicate the significantly down-regulated and up-regulated DEGs, respectively. **(B)** Heatmap of correlation between ERVFRD-1 expression and the top 10 DEGs. **(C)** Top 10 hub genes in ERVFRD-1 expression-associated DEGs. Red to yellow, higher to lower order. **(D)** GO and KEGG analysis of DEGs. GO, Gene Ontology; KEGG, Kyoto Encyclopedia of Genes and Genomes; DEGs, differentially expressed genes.

Through a comparison of gene expression profiles between KIRC samples with high and low ERVFRD-1 levels, we discovered a total of 1348 DEGs, which consisted of 128 (9.50%) upregulated genes and 1220 (90.50%) downregulated genes (adjusted p-value < 0.05, |Log2-FC| > 1) (**Figure 3A**). We further explored the relationship between the top 10 DEGs (including AC090578.1, PAEP, FDCSP, SAA1, GOLGA6L7, APOA4, IGFBP1, RTL1, HHATL, and NMRK2) and ERVFRD-1, as illustrated in **Figure 3B** and **Supplementary Table S2**. We employed the online STRING tool to construct a PPI network that aimed to investigate the potential interactions within the DEGs (**Supplementary Figure S1**). Moreover, we identified a group of hub genes from the PPI network using the same tool. The network displayed a high level of complexity, and the top 10 hub genes were APOB, AHSG, FGA, FGG, FGB, APOC3, APOA4, IVL, SPRR1B, and RPTN, as shown in **Figure 3C**.

### 3.5 Functional enrichment analysis including GO, KEGG, and GSEA analysis

We then conducted GO and KEGG enrichment analyses to gain insights into the biological functions of the abovementioned DEGs. GO analysis revealed that these DEGs were primarily involved in “humoral immune response” and “complement activation, classical pathway” in terms of biological processes (GO-BP). In terms of cellular components (GO-CC), the DEGs were enriched in “immunoglobulin complex” and “immunoglobulin complex.” The molecular function (MF) analysis showed enrichment in “antigen binding” and “immunoglobulin receptor binding.” Additionally, the KEGG pathway analysis demonstrated enrichment in “neuroactive ligand-receptor interaction” and “complement and coagulation cascades.” These findings are depicted in **Figure 3D** and **Supplementary Table S3**.

**Figure 4:**
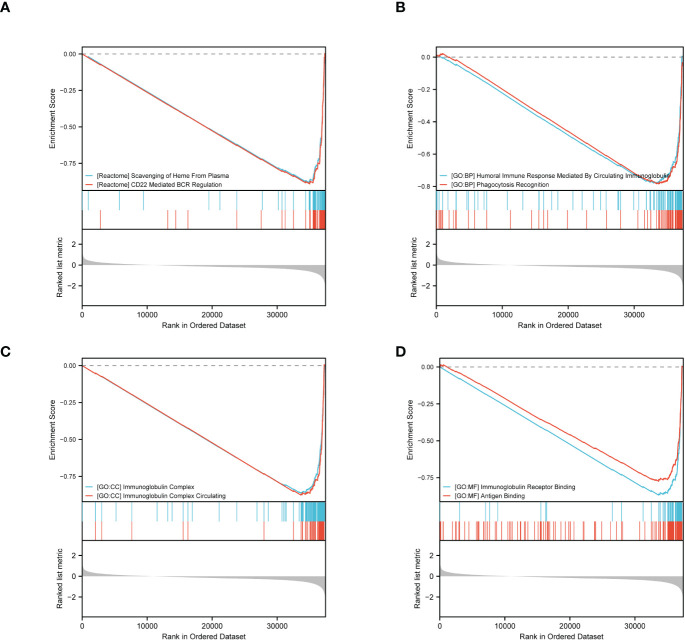
GSEA of ERVFRD-1 related DEGs. GSEA analysis of all canonical pathways gene sets **(A)**, bp **(B)**, cc **(C)** and mf **(D)** of GO genes deposited or downloaded from MSigDB. GSEA, gene set enrichment analysis; DEGs, differentially expressed genes; bp, biological process; mf for molecular function; cc for cellular component; GO, Gene Ontology; MSigDB, Molecular Signatures database; NES, normalized enrichment score.

Furthermore, we performed GSEA using GSEA/Molecular Signatures Database (MSigDB), which revealed significant enrichment in immune-related biological processes, including humoral immune response, immunoglobulin complex, and immunoglobulin receptor binding, among others. These results suggest that ERVFRD-1 may contribute to an enhanced immune phenotype in KIRC (**Figure 4**; **Supplementary Table S4**).

### 3.6 Correlation between ERVFRD-1 expression and immune infiltration in KIRC

**Figure 5:**
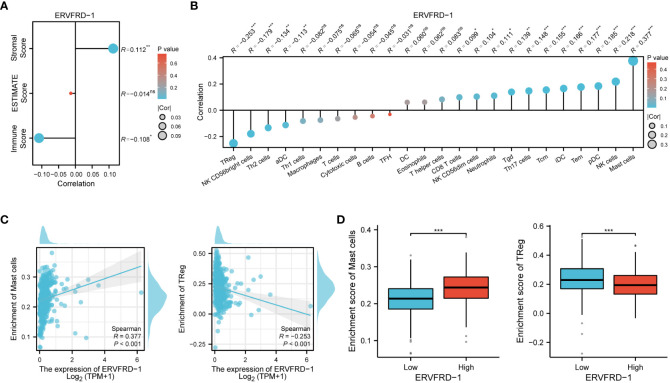
Correlation of ERVFRD-1 expression with immune infiltration level in KIRC. **(A)** Correlation between ERVFRD-1 expression and stromal score, immunescore and ESTIMATE score in KIRC. **(B)** Correlation between ERVFRD-1 expression and relative abundance of 24 types of immune cell. The size of dot corresponds to the absolute Spearman’s correlation coefficient values. **(C)** Correlations between the relative enrichment scores of immune cells (including mast cells and Treg cells) and expression of ERVFRD-1. **(D)** Comparison of immune infiltration levels of immune cells (including mast cells and Treg cells) between high- and low-ERVFRD-1 expression groups. TReg cells, Regulatory T cells. ns, not significant; *P < 0.05, **P < 0.01, ***P < 0.001.

To investigate the correlation between ERVFRD-1 expression and immune infiltrates in KIRC, we utilized the ESTIMATE algorithm to determine stromal scores, immune scores, and ESTIMATE scores. Our findings indicate that ERVFRD-1 expression is significantly and positively correlated with stromal score, but negatively correlated with immune score, suggesting a notable impact on stromal and immune cell infiltration (as shown in **Figure 5A**). Then we utilized ssGSEA and found a correlation between ERVFRD-1 expression and the levels of immune infiltrating cells, specifically mast cells (r=0.377, P < 0.001) and Regulatory T cells (Treg, r = -0.253, P < 0.001) (**Figure 5B**). The enrichment scores for mast cells were significantly higher in the group with high expression of ERVFRD-1 compared to the group with low expression, while Treg cells exhibited the opposite trend (all P < 0.001), as depicted in **Figures 5C, D**.

**Figure 6:**
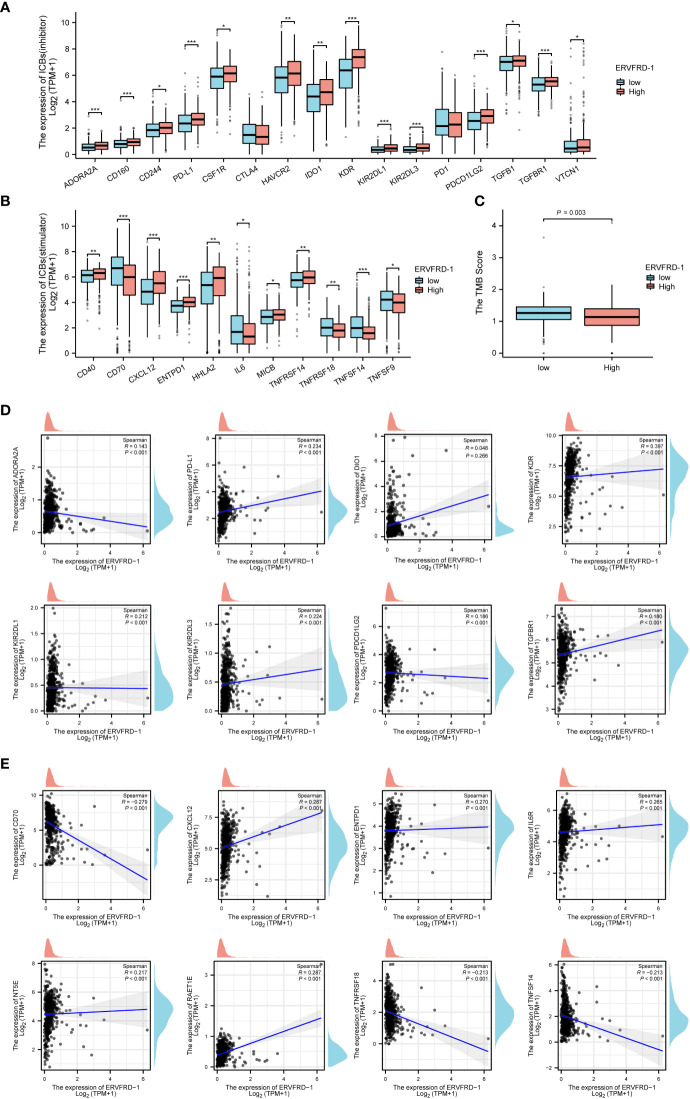
Correlation of ERVFRD-1 expression with immune biomarkers in KIRC. **(A)** Correlations between ERVFRD-1 expression and immunoinhititors. **(B)** Correlations between ERVFRD-1 expression and immunostimulators. **(C)** Correlations between relative TMB scores and ERVFRD-1 expression. **(D)** Scattered plots showed correlations, with significance difference, between ERVFRD-1 expression and immunoinhititors including ADORA2A, PD-L1, DIO1, KDR, KIR2DL1, KIR2DL3, PDCD1LG2 and TGFBR1, and **(E)** immunostimulators including CD70, CXCL12, ENTPD1, IL6R, NT5E, RAET1E, TNFRSF18, and TNFRSF14. *P < 0.05, **P < 0.01, ***P < 0.001.

Additionally, we investigated the correlation between ERVFRD-1 expression and immune biomarkers, including immune modulators and tumor mutational burden (TMB). Our analysis revealed that the expression of ERVFRD-1 demonstrated a significant positive correlation with immunoinhibitors and most immunostimulators (e.g., CD70 and TNFSF14) (**Figures 6A, B**). In terms of TMB, higher TMB scores were observed in the low-expression group of ERVFRD-1, indicating the lower level of ERVFRD-1 was more likely to benefit from immunotherapy (P = 0.003) (**Figure 6C**). Additionally, a series of scattered plots, as depicted in **Figures 6D, E**, displayed the correlation between ERVFRD-1 with a few immunoinhibitors (ADORA2A, PD-L1, DIO1, KDR, KIR2DL1, KIR2DL3, PDCD1LG2, and TGFBR1) and immunostimlators (CD70, CXCL12, ENTPD1, IL6R, NT5E, RAET1E, TNFRSF18, and TNFRSF14). Furthermore, we examined 22 KIRC patients who had received immunotherapy (PD-L1/PD-1) from TCGA database and analyzed their response based on ERVFRD-1 expression levels. However, our results suggest no significant differences between high and low ERVFRD-1 expressing groups (**Supplementary Figure S2**).

### 3.7 Correlation between methylation and expression of ERVFRD-1 in KIRC

**Figure 7:**
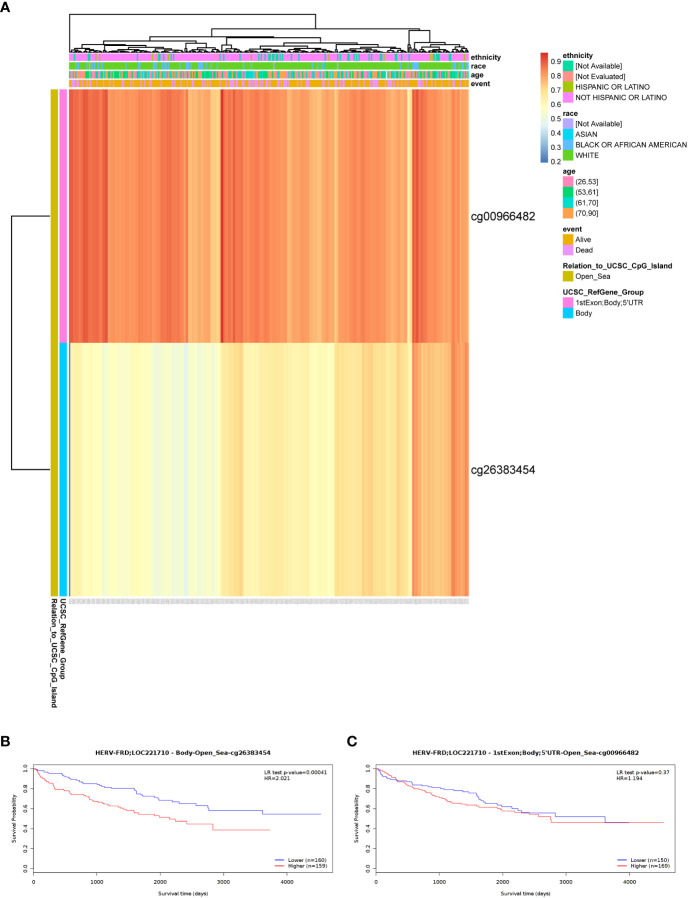
DNA methylation level of ERVFRD-1 and its effect on prognosis of KIRC patients. **(A)** Correlation between KIRC mRNA expression level and methylation level. Two probes, cg00966482 and cg26383454, detected methylation positions on the ERVFRD-1 gene. cg00966482 is located in “1stExon; Body; 5’UTR; Open_Sea” regions. Correlation results indicate elevated methylation levels in these positions (depicted in reddish colors). cg26383454 is located in the “Body; open_sea” region, and the correlation results suggest moderately elevated methylation levels (shown in lighter shades of yellow). **(B)** Lower methylation levels in the Body-open_sea region of ERVFRD-1 gene correlate with better overall survival prognosis. **(C)** Methylation in the “1stExon; Body; 5’UTR; Open_Sea” regions of ERVFRD-1 gene is not associated with overall survival prognosis.

To shed light on the underlying mechanisms of ERVFRD-1 overexpression in KIRC tissues, we utilized the online tool “MethSurv database” to investigate the correlation between ERVFRD-1 expression levels and methylation status. Our analysis revealed that the DNA sequences of ERVFRD-1 contain two single Cytosine-phosphate-Guanine (CpG) sites, cg00966482 and cg26383454 (**Figure 7A**). Both of these methylation sites were observed to be methylated in KIRC, especially cg00966482. Furthermore, we identified patients who exhibited high levels of ERVFRD-1 methylation in the cg26383454 site had poorer overall survival rates when compared to those with lower levels of ERVFRD-1 methylation, while the methylation status of the cg00966482 site did not show any significant difference in survival between the high and low groups (**Figures 7B, C**).

### 3.8 Prognostic value of ERVFRD-1 in KIRC

**Figure 8:**
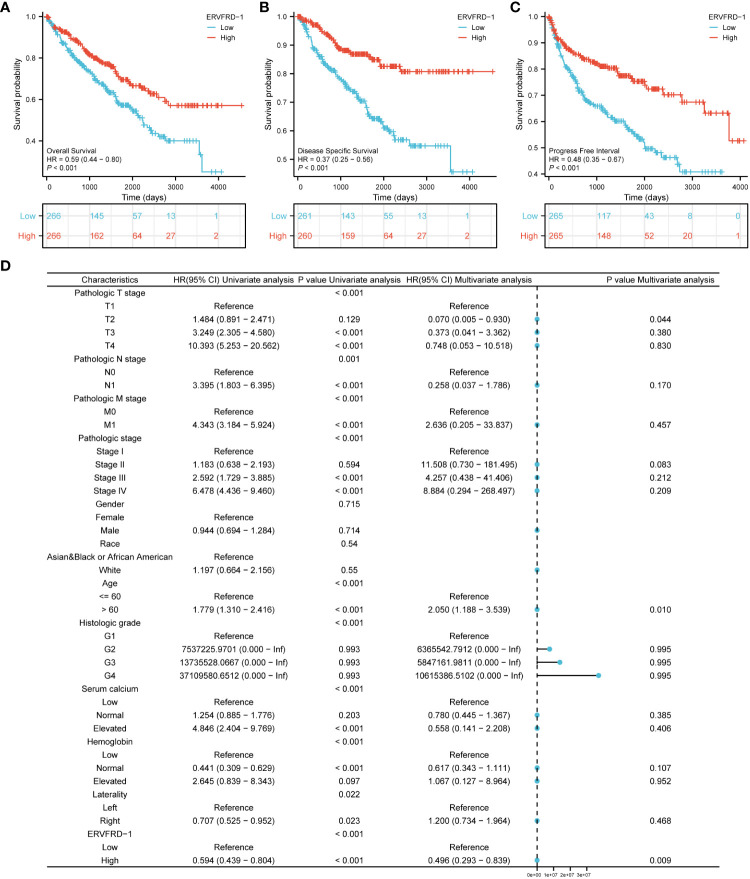
Prognostic values of ERVFRD-1 in patients with KIRC. **(A–C)** Kaplan-Meier analysis of overall survival, disease-specific survival, progress free interval in KIRC patients with high vs low expression of ERVFRD-1. **(D)** Forest map based on multivariate Cox analysis for overall survival. ROC, receiver operating characteristic; HR, hazard ratio; CI, confidence interval.

We conducted survival analysis using the Kaplan-Meier method to evaluate the association between ERVFRD-1 expression and the survival outcomes of patients with KIRC. Patients were divided into high and low expression groups based on the median value of ERVFRD-1 expression. The high ERVFRD-1 expression group displayed a significantly better prognosis for overall survival (OS), disease-specific survival (DSS), and progression-free interval (PFI) in comparison to the low expression group (OS: hazard ratio [HR] = 0.59, 95% CI = 0.44 – 0.80, P < 0.001; DSS: HR = 0.37, 95% CI = 0.25 – 0.56, P < 0.001; PFI: HR = 0.48, 95% CI = 0.35 – 0.67, P < 0.001), as demonstrated by the corresponding Kaplan-Meier curves (**Figures 8A–C**).

Furthermore, we performed univariate and multivariate Cox regression analyses to identify prognostic factors. The multivariate analysis revealed that ERVFRD-1 expression (adjusted HR = 0.496, 95% CI = 0.293-0.839, P = 0.009), T2 stage (adjusted HR = 0.070, 95% CI = 0.005-0.935, P = 0.044), and age stage (adjusted HR = 2.050, 95% CI = 1.188-3.539, P = 0.010) were independent factors associated with OS in patients with KIRC (**Figure 8D**; **Supplementary Table S5**). Similarly, for DSS, ERVFRD-1 expression (adjusted HR = 0.375, 95% CI = 0.183 - 0.770, P = 0.008) was identified as a prognostic indicator (**Supplementary Table S6**). However, for PFI, ERVFRD-1 expression did not show prognostic significance (**Supplementary Table S7**).

**Figure 9:**
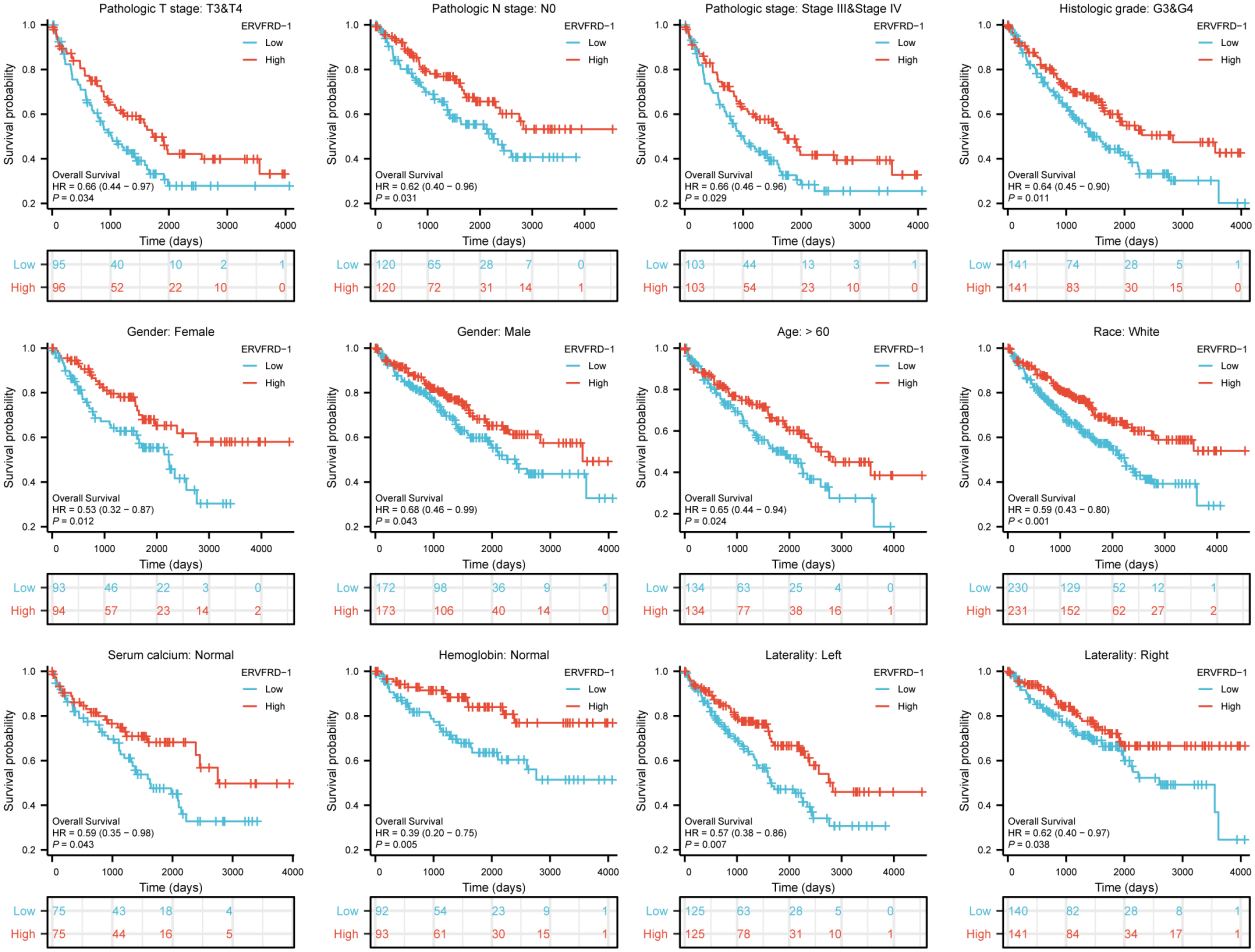
Prognostic values of ERVFRD-1 expression in patients with KIRC evaluated by Kaplan-Meier analysis in different clinical subgroups. OS survival curves of T3-4, N0, stage III-IV, grade 3&4, female, male, age >60 years, white race, normal serum calcium, normal hemoglobin, left and right laterality.

We further assessed the prognostic value of ERVFRD-1 expression in different subgroups. Consistently, high expression of ERVFRD-1 was associated with favorable outcomes in various subgroups based on OS, including T3 & T4 stage, N0 stage, stages III & IV, grade 3& 4, gender, age > 60 years, white race, normal serum calcium, normal hemoglobin, and laterality (all P < 0.05) (**Figure 9**).

### 3.9 Construction and validation of a nomogram based on independent factors

**Figure 10:**
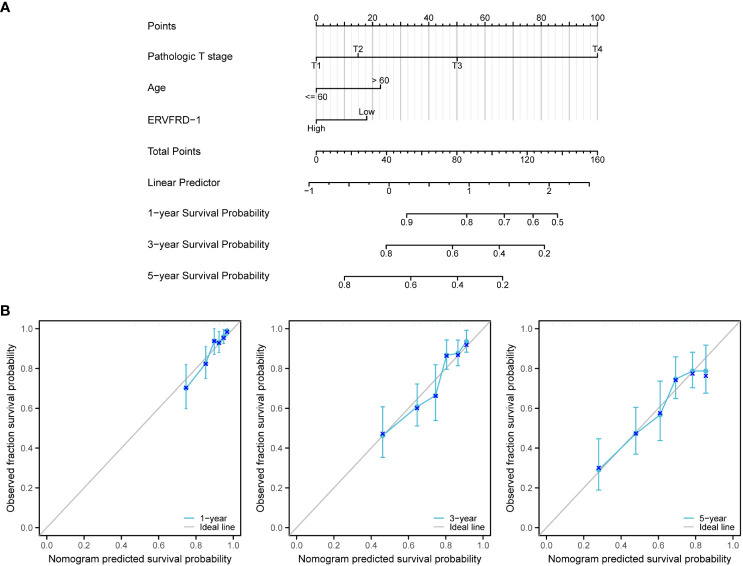
A nomogram and calibration curves to predict 1-, 3-, and 5-year overall survival rates of patients with KIRC. **(A)** A nomogram for prediction of 1-, 3-, and 5-year overall survival rates of patients with KIRC. **(B)** Calibration curves of the nomogram prediction of 1-, 3-, and 5-year overall survival rates of patients with KIRC.

We developed a nomogram using the independent prognostic factors for OS to provide a prognostic prediction tool for KIRC patients. The nomogram visually represented the total points assigned to each factor, where a higher total number of points indicated a worse prognosis (**Figure 10A**). Calibration curves were generated to assess the performance of the nomogram in predicting survival outcomes, demonstrating good agreement between predicted and observed survival probabilities (**Figure 10B**). The bootstrap corrected C-index of the nomogram was 0.710 (95% CI = 0.690 - 0.730), indicating a moderate predictive accuracy for OS in patients with KIRC. Additionally, we assessed the discriminative ability of ERVFRD-1 expression using time-dependent ROC curve analysis (**Supplementary Figure S3**). In summary, these observations suggested that the nomogram was an appropriate tool for predicting the prognosis of KIRC patients.

## 4 Discussion

Kidney renal cell carcinoma (KIRC), characterized by a microenvironment that is angiogenic, inflammatory, and highly immunogenic, has limited response to chemotherapy and radiation therapy (Kim et al., 2021). However, recent studies have shown the efficacy of immunotherapy as a treatment option for KIRC(Choueiri et al., 2020; Powles et al., 2022), underscoring the significance of investigating molecular markers that can provide prognostic information and insights into the immune response in this disease. The HERV family, remnants of ancient viruses, may not exhibit viral activity at the transcriptional level but has been found to enhance immune antigenicity. This characteristic has also been observed in tumor immunotherapy, making the exploration of HERVs crucial in understanding their role in the context of KIRC.

Evidence from previous publications has indicated abnormal expression of certain HERVs in urinary tract malignancies. For instance, upregulation of Syncytin-1, encoded by ERVW-1, is more frequently observed in bladder urothelial cancer compared to healthy tissues (Yu et al., 2014). A study by Veronika Weyerer and colleagues found significantly higher levels of expression for several HERVs, including Syncytin-1, ERV-H, ERV-Fc1, ERV-Fc2, ERV-T, ERV-Kenv, ERV-Kpol, ERV-E4-1, and ERV-E6q15 in KIRC lesions when compared to normal kidney samples (Weyerer et al., 2021). Our study, for the first time, provides direct evidence of a potentially vital role regarding the expression of ERVFRD-1 in KIRC. ERVFRD-1 is typically expressed in the placenta and thought to protect the fetus from rejection by the mother (Bolze et al., 2017). To begin with, our findings revealed from TCGA database that ERVFRD-1 exhibited low expression levels in KIRC tissues compared to normal tissues. Of note, KIRC patients with elevated expression of ERVFRD-1 had favorable survival. The multivariate Cox analysis demonstrated that T stage, age, and ERVFRD-1 expression possessed independent prognostic values in KIRC. Collectively, these results demonstrate that ERVFRD-1 holds promise as an attractive and novel prognostic biomarker for KIRC.

To better understand the role of ERVFRD-1 in KIRC, we conducted further investigations into its biological function and signaling pathways. The exploration of differentially expressed genes (DEGs) using GO enrichment and KEGG pathway analyses revealed several immune-related pathways and functions associated with ERVFRD-1. It has been previously demonstrated that epigenetically silenced HERVs can become transcriptionally activated in different cancers, resulting in adaptive immune responses to HERV epitopes (Cherkasova et al., 2013; Downey et al., 2015). Spontaneous T-cell and B-cell responses against HERV antigens are also well-documented (Ebert et al., 2009). Previous studies also suggested ERVFRD-1 as an essential immune regulator, both locally and systemically, through its association with placental exosomes (Lokossou et al., 2020). However, further investigation is required to fully understand the immune regulation of ERVFRD-1 in the context of KIRC.

The tumor microenvironment (TME) is composed of tumor cells and various non-malignant cells, including tumor-infiltrating immune cells (TIICs), fibroblasts, and stromal cells (Wu and Dai, 2017; Zheng et al., 2021). Among these, TIICs have been identified as major regulators of tumor development at different stages (Lei et al., 2020). Building upon our previous findings, we further investigated the relationship between ERVFRD-1 and TIICs in KIRC using GSEA. Our study revealed a positive correlation between ERVFRD-1 expression and mast cells, while a negative correlation was observed with Tregs.

Moreover, previous studies have suggested that the infiltration of dendritic cell resting, mast cell resting, and eosinophilia in KIRC is associated with a favorable prognosis (Pan et al., 2020). Conversely, Tregs are known to be highly immunosuppressive towards effector cells and their increased infiltration during KIRC progression is indicative of a poor prognosis (Şenbabaoğlu et al., 2016; Braun et al., 2021). Additionally, the degree of Treg infiltration is lower in some patients who respond favorably to immune checkpoint inhibitors (ICI) and achieve complete response (Krishna et al., 2021). Taken together, our finding on the prognostic role of ERVFRD-1 supports these previous observations, given its protective effect on overall survival and longer survival outcome.

Checkpoint inhibition therapies targeting PD-L1 or CTLA-4 pathways have shown promising effects in patients with renal cell carcinoma (Choueiri et al., 2016; Motzer et al., 2018). A recent study revealed that responders to these therapies exhibited significantly higher expression of ERV3-2 in tumors compared to non-responders (Panda et al., 2018). Additionally, a TCGA bioinformatics profiling of 18 different cancer types identified specific associations between KIRC and ERV-E, as well as seven different ERV-K families (Rooney et al., 2015). Furthermore, ERV-E env has been identified as a potential target for T-cell-based immunotherapy, specifically in KIRC (Cherkasova et al., 2016). Interestingly, our findings demonstrated a positive correlation between ERVFRD-1 and PD-L1 expression, but not with PD-1 or CTLA-4. Notably, KDR (a gene encoding the VEGF receptor) was found to be the most correlated factor with ERVFRD-1 in our analysis.

In the absence of further supporting evidence, our study aimed to explore the possible relationship between the expression of ERVFRD-1 and immunotherapy in KIRC. Common immunotherapy indices include tumor mutation burden (TMB) and immune checkpoint expression. We aimed to investigate whether ERVFRD-1 has therapeutic value in KIRC immunotherapy by comparing its expression patterns with that of immune checkpoints. Our findings reveal a weak yet statistically significant correlation between TMB and PD-L1 with ERVFRD-1 expression. Next, we sought to determine whether ERVFRD-1 expression has a prognostic value in immunotherapy response in the KIRC population from TCGA dataset. Although our findings did not display a response to PD-L1/PD-1, we must exercise caution in interpreting these results due to our limited sample size, which highlights the need for further validation studies with larger sample sizes. Furthermore, our analysis found that ERVFRD-1 exhibited a higher correlation with other immune stimulators such as CD70 and TNFSF14. These findings suggest that ERVFRD-1 may play a significant role in activating the immune system to achieve an anti-tumor effect in KIRC.

Last but not least, a recent article has reported that the DNA hypomethylating agent decitabine can activate the expression of transposable elements (TEs) such as LINE1, ERV3-2, and ERV4700, as well as antiviral signaling, to enhance the response to immune checkpoint blockade (ICB) in KIRC and primary cells (de Cubas et al., 2020). Thus, targeting ERVs through methylation regulation may represent another potential therapeutic approach for cancer treatment. In light of this, we also investigated the methylation status of ERVFRD-1 in KIRC and found that methylation predominantly drives ERVFRD-1 expression. Additionally, we observed higher levels of methylation in the cg26383454 site were associated with a poor prognosis. Therefore, the use of demethylating agents may also be a viable strategy for improving the prognosis of KIRC patients with high ERVFRD-1 methylation.

## 5 Conclusions

In summary, this study investigated the expression and potential prognostic value of ERVFRD-1 in patients with KIRC. Through comprehensive analyses of clinical and molecular data from various databases, the study revealed a correlation between ERVFRD-1 expression and immune infiltrates, as well as immune modulators in KIRC. GO and KEGG enrichment analysis revealed that ERVFRD-1 may increase the immune phenotype in KIRC. Furthermore, high ERVFRD-1 expression was associated with a favorable prognostic factor for OS, DSS, and PFI in KIRC patients. Multivariate Cox regression analysis identified ERVFRD-1 expression as an independent prognostic factor for overall survival. A nomogram incorporating ERVFRD-1 expression and other clinical variables was developed to predict patient outcomes, demonstrating moderate predictive accuracy. The findings highlight the potential significance of ERVFRD-1 as a prognostic biomarker and provide a valuable tool for personalized prognosis assessment in KIRC patients.

## Data availability statement

The datasets presented in this study can be found in online repositories. The names of the repository/repositories and accession number(s) can be found in the article/**Supplementary Material**.

## Author contributions

XW, JS, and LS contributed to the conception and design of the study. XF and JX both analyzed and interpreted the data using different algorithms, structured and drafted the original manuscript. MDM and DZ re-analyzed the data, reviewed, and edited the manuscript. LS supervised all aspects of the study. All authors contributed to the article and approved the submitted version.
