# Supplementary material for: Endogenous retrovirus group FRD member 1 is a potential biomarker for prognosis and immunotherapy for kidney renal clear cell carcinoma
Source: Front Cell Infect Microbiol. 2023 Sep 13;13:1252905. doi: 10.3389/fcimb.2023.1252905 (PMC10534008; doi:10.3389/fcimb.2023.1252905)
Supplement: Supplementary file 2 [file Image_2.pdf]

Supplementary Figure S2

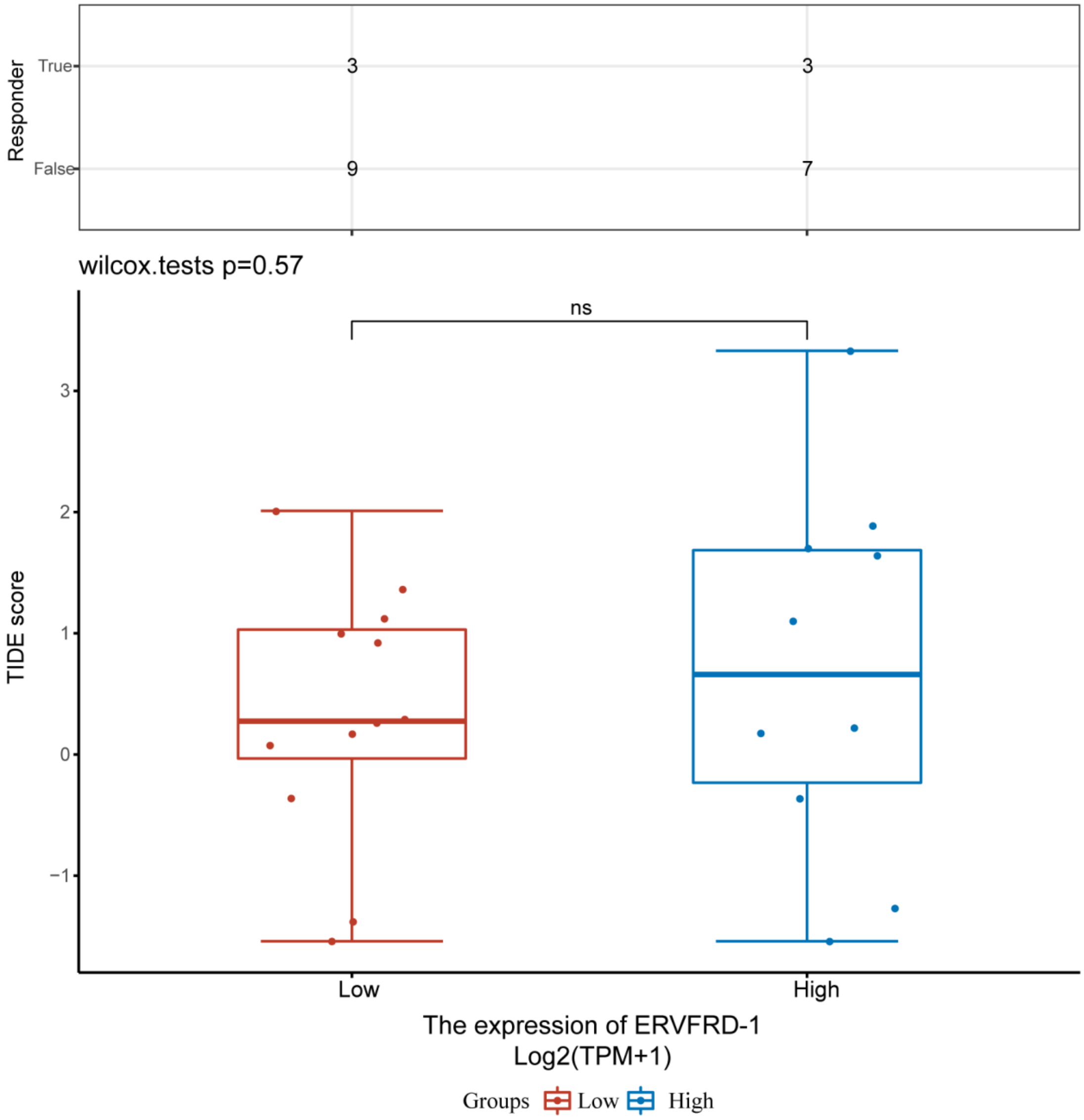

Supplementary figure S2. Response to PD-L1/PD-1 immunotherapy based on ERVFRD-1 expression levels. Up: Statistical table of immune response of samples in ERVFRD-1 low-high expression groups. Down: The distribution of immune response scores in ERVFRD-1 low-high expression groups.
