# Supplementary material for: Endogenous retrovirus group FRD member 1 is a potential biomarker for prognosis and immunotherapy for kidney renal clear cell carcinoma
Source: Front Cell Infect Microbiol. 2023 Sep 13;13:1252905. doi: 10.3389/fcimb.2023.1252905 (PMC10534008; doi:10.3389/fcimb.2023.1252905)
Supplement: Supplementary file 3 [file Image_3.pdf]

Supplementary Figure S3

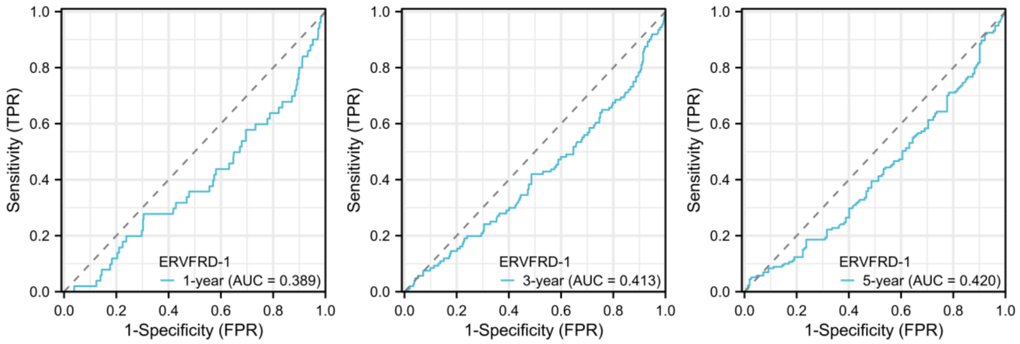

Supplementary figure S3. Time-dependent ROC curves at different time points.

Time-dependent ROC curves based on ERVFRD-1 expression for one-, three-, and five-year OS probability. ROC, receiver-operating characteristic; AUC, area under the ROC curve; OS, overall survival.
