# Supplementary material for: Endogenous retrovirus group FRD member 1 is a potential biomarker for prognosis and immunotherapy for kidney renal clear cell carcinoma
Source: Front Cell Infect Microbiol. 2023 Sep 13;13:1252905. doi: 10.3389/fcimb.2023.1252905 (PMC10534008; doi:10.3389/fcimb.2023.1252905)
Supplement: Supplementary file 4 [file Table_1.docx]

Supplementary Table S1

Baseline characteristics of patients of KIRC (n = 532).

| Characteristics | Overall |
| --- | --- |
| Age, median (IQR) | 61 (52, 70) |
| Pathologic T stage, n (%) |  |
| T1 | 272 (51.1%) |
| T2 | 69 (13%) |
| T3 | 180 (33.8%) |
| T4 | 11 (2.1%) |
| Pathologic N stage, n (%) |  |
| N0 | 240 (93.8%) |
| N1 | 16 (6.2%) |
| Pathologic M stage, n (%) |  |
| M0 | 421 (84.2%) |
| M1 | 79 (15.8%) |
| Pathologic stage, n (%) |  |
| Stage I | 266 (50.3%) |
| Stage II | 57 (10.8%) |
| Stage III | 123 (23.3%) |
| Stage IV | 83 (15.7%) |
| Gender, n (%) |  |
| Female | 187 (35.2%) |
| Male | 345 (64.8%) |
| Race, n (%) |  |
| Asian&Black or African American | 64 (12.2%) |
| White | 461 (87.8%) |
| Age, n (%) |  |
| <= 60 | 264 (49.6%) |
| > 60 | 268 (50.4%) |
| Histologic grade, n (%) |  |
| G1 | 14 (2.7%) |
| G2 | 228 (43.5%) |
| G3 | 206 (39.3%) |
| G4 | 76 (14.5%) |
| Serum calcium, n (%) |  |
| Low | 204 (56%) |
| Normal | 150 (41.2%) |
| Elevated | 10 (2.7%) |
| Hemoglobin, n (%) |  |
| Low | 262 (58%) |
| Normal | 185 (40.9%) |
| Elevated | 5 (1.1%) |
| Laterality, n (%) |  |
| Left | 250 (47.1%) |
| Right | 281 (52.9%) |
| OS event, n (%) |  |
| Alive | 357 (67.1%) |
| Dead | 175 (32.9%) |
| DSS event, n (%) |  |
| Alive | 412 (79.1%) |
| Dead | 109 (20.9%) |
| PFI event, n (%) |  |
| No | 371 (69.7%) |
| Yes | 161 (30.3%) |

Abbreviations: KIRC, Kidney Renal Clear Cell Carcinoma; IQR, Interquartile Range.
