# Supplementary material for: Endogenous retrovirus group FRD member 1 is a potential biomarker for prognosis and immunotherapy for kidney renal clear cell carcinoma
Source: Front Cell Infect Microbiol. 2023 Sep 13;13:1252905. doi: 10.3389/fcimb.2023.1252905 (PMC10534008; doi:10.3389/fcimb.2023.1252905)
Supplement: Supplementary file 5 [file Table_2.docx]

Supplementary Table S2

Top 10 *ERVFRD-1* related DEGs.

| gene_name | id | baseMean | log2FoldChange | lfcSE | stat | pvalue | padj | gene_type |
| --- | --- | --- | --- | --- | --- | --- | --- | --- |
| AC090578.1 | ENSG00000253553.7 | 79.5551099 | -3.96886881 | 0.2265902 | -17.5156 | 1.09E-68 | 2.36E-64 | lncRNA |
| PAEP | ENSG00000122133.17 | 131.360628 | -3.92838323 | 0.3007564 | -13.0617 | 5.45E-39 | 1.69E-35 | protein_coding |
| FDCSP | ENSG00000181617.6 | 42.0752138 | -3.91589721 | 0.3368476 | -11.6251 | 3.07E-31 | 5.13E-28 | protein_coding |
| SAA1 | ENSG00000173432.12 | 3634.35752 | -3.6609838 | 0.266695 | -13.7272 | 6.97E-43 | 3.78E-39 | protein_coding |
| GOLGA6L7 | ENSG00000261649.6 | 36.9649782 | -3.59426422 | 0.2319039 | -15.4989 | 3.53E-54 | 5.1E-50 | protein_coding |
| APOA4 | ENSG00000110244.7 | 2.71241058 | -3.55330161 | 0.5671556 | -6.26513 | 3.73E-10 | 8.86E-09 | protein_coding |
| IGFBP1 | ENSG00000146678.10 | 2153.69132 | -3.54814386 | 0.2427588 | -14.6159 | 2.22E-48 | 1.93E-44 | protein_coding |
| RTL1 | ENSG00000254656.3 | 10.624192 | -3.50091223 | 0.317743 | -11.0181 | 3.13E-28 | 4.11E-25 | protein_coding |
| HHATL | ENSG00000010282.15 | 66.6537738 | 3.44598395 | 0.3142336 | 10.96631 | 5.55E-28 | 6.66E-25 | protein_coding |
| NMRK2 | ENSG00000077009.13 | 57.6298407 | 3.43819831 | 0.2944791 | 11.67553 | 1.7E-31 | 3.21E-28 | protein_coding |

Abbreviations: DEGs, differentially expressed genes; p.adj, adjusted *p* value.
