# Supplementary material for: Endogenous retrovirus group FRD member 1 is a potential biomarker for prognosis and immunotherapy for kidney renal clear cell carcinoma
Source: Front Cell Infect Microbiol. 2023 Sep 13;13:1252905. doi: 10.3389/fcimb.2023.1252905 (PMC10534008; doi:10.3389/fcimb.2023.1252905)
Supplement: Supplementary file 6 [file Table_3.docx]

Supplementary Table S3

GO and KEGG pathway functional enrichment for ERVFRD-1 related DEGs.

| ONTOLOGY | ID | Description |
| --- | --- | --- |
| BP | GO:0006959 | humoral immune response |
| BP | GO:0006910 | phagocytosis, recognition |
| BP | GO:0006958 | complement activation, classical pathway |
| BP | GO:0002455 | humoral immune response mediated by circulating immunoglobulin |
| CC | GO:0019814 | immunoglobulin complex |
| CC | GO:0042571 | immunoglobulin complex, circulating |
| CC | GO:0072562 | blood microparticle |
| CC | GO:0009897 | external side of plasma membrane |
| MF | GO:0003823 | antigen binding |
| MF | GO:0034987 | immunoglobulin receptor binding |
| MF | GO:0030280 | structural constituent of skin epidermis |
| MF | GO:0004866 | endopeptidase inhibitor activity |
| KEGG | hsa04080 | Neuroactive ligand-receptor interaction |
| KEGG | hsa04610 | Complement and coagulation cascades |
| KEGG | hsa04950 | Maturity onset diabetes of the young |
| KEGG | hsa04975 | Fat digestion and absorption |

Abbreviations: GO, Gene Ontology; KEGG, Kyoto Encyclopedia of Genes and Genomes; DEGs, differentially expressed genes; BP, biological processes; CC, subcellular localizations; MF, molecular functions.
