# Supplementary material for: Endogenous retrovirus group FRD member 1 is a potential biomarker for prognosis and immunotherapy for kidney renal clear cell carcinoma
Source: Front Cell Infect Microbiol. 2023 Sep 13;13:1252905. doi: 10.3389/fcimb.2023.1252905 (PMC10534008; doi:10.3389/fcimb.2023.1252905)
Supplement: Supplementary file 7 [file Table_4.docx]

Supplementary Table S4

Gene set enrichment analysis for *ERVFRD-1* related DEGs.

| ID | setSize | enrichmentScore | NES | pvalue | p.adjust | qvalue | rank | leading_edge |  |
| --- | --- | --- | --- | --- | --- | --- | --- | --- | --- |
| REACTOME_SCAVENGING_OF_HEME_FROM_PLASMA | | 69 | -0.88201 | -2.75308 | 1E-10 | 9.97E-09 | 8.33E-09 | 2519 | tags=83%, list=7%, signal=77% |
| REACTOME_CD22_MEDIATED_BCR_REGULATION | | 61 | -0.89133 | -2.74498 | 1E-10 | 9.97E-09 | 8.33E-09 | 2442 | tags=84%, list=7%, signal=78% |
| GOBP_HUMORAL_IMMUNE_RESPONSE_MEDIATED_BY_CIRCULATING_IMMUNOGLOBULIN | | 111 | -0.78049 | -2.52415 | 1E-10 | 2E-08 | 1.66E-08 | 4030 | tags=68%, list=11%, signal=60% |
| GOBP_PHAGOCYTOSIS_RECOGNITION | | 95 | -0.78468 | -2.50922 | 1E-10 | 2E-08 | 1.66E-08 | 3779 | tags=69%, list=10%, signal=63% |
| GOCC_IMMUNOGLOBULIN_COMPLEX | | 155 | -0.86318 | -2.87607 | 1E-10 | 1.59E-08 | 1.4E-08 | 3779 | tags=85%, list=10%, signal=77% |
| GOCC_IMMUNOGLOBULIN_COMPLEX_CIRCULATING | | 68 | -0.87728 | -2.70979 | 1E-10 | 1.59E-08 | 1.4E-08 | 3779 | tags=90%, list=10%, signal=81% |
| GOMF_IMMUNOGLOBULIN_RECEPTOR_BINDING | | 71 | -0.86848 | -2.73876 | 1E-10 | 5.47E-08 | 4.64E-08 | 3779 | tags=85%, list=10%, signal=76% |
| GOMF_ANTIGEN_BINDING | | 165 | -0.7747 | -2.59678 | 1E-10 | 5.47E-08 | 4.64E-08 | 3780 | tags=56%, list=10%, signal=51% |

Abbreviations: GSEA, gene set enrichment analysis; BP, biological processes; CC, subcellular localizations; MF, molecular functions.
