# Supplementary material for: Endogenous retrovirus group FRD member 1 is a potential biomarker for prognosis and immunotherapy for kidney renal clear cell carcinoma
Source: Front Cell Infect Microbiol. 2023 Sep 13;13:1252905. doi: 10.3389/fcimb.2023.1252905 (PMC10534008; doi:10.3389/fcimb.2023.1252905)
Supplement: Supplementary file 8 [file Table_5.docx]

Supplementary Table S5

Univariate and multivariate analyses of overall survival in patients with KIRC.

| Characteristics | Total(N) | HR(95% CI) Univariate analysis | P value Univariate analysis | HR(95% CI) Multivariate analysis | P value Multivariate analysis |
| --- | --- | --- | --- | --- | --- |
| Pathologic T stage | 532 |  | < 0.001 |  |  |
| T1 | 272 | Reference |  | Reference |  |
| T2 | 69 | 1.484 (0.891 - 2.471) | 0.129 | 0.070 (0.005 - 0.930) | 0.044 |
| T3 | 180 | 3.249 (2.305 - 4.580) | < 0.001 | 0.373 (0.041 - 3.362) | 0.380 |
| T4 | 11 | 10.393 (5.253 - 20.562) | < 0.001 | 0.748 (0.053 - 10.518) | 0.830 |
| Pathologic N stage | 256 |  | 0.001 |  |  |
| N0 | 240 | Reference |  | Reference |  |
| N1 | 16 | 3.395 (1.803 - 6.395) | < 0.001 | 0.258 (0.037 - 1.786) | 0.170 |
| Pathologic M stage | 500 |  | < 0.001 |  |  |
| M0 | 421 | Reference |  | Reference |  |
| M1 | 79 | 4.343 (3.184 - 5.924) | < 0.001 | 2.636 (0.205 - 33.837) | 0.457 |
| Pathologic stage | 529 |  | < 0.001 |  |  |
| Stage I | 266 | Reference |  | Reference |  |
| Stage II | 57 | 1.183 (0.638 - 2.193) | 0.594 | 11.508 (0.730 - 181.495) | 0.083 |
| Stage III | 123 | 2.592 (1.729 - 3.885) | < 0.001 | 4.257 (0.438 - 41.406) | 0.212 |
| Stage IV | 83 | 6.478 (4.436 - 9.460) | < 0.001 | 8.884 (0.294 - 268.497) | 0.209 |
| Gender | 532 |  | 0.715 |  |  |
| Female | 187 | Reference |  |  |  |
| Male | 345 | 0.944 (0.694 - 1.284) | 0.714 |  |  |
| Race | 525 |  | 0.540 |  |  |
| Asian&Black or African American | 64 | Reference |  |  |  |
| White | 461 | 1.197 (0.664 - 2.156) | 0.550 |  |  |
| Age | 532 |  | < 0.001 |  |  |
| <= 60 | 264 | Reference |  | Reference |  |
| > 60 | 268 | 1.779 (1.310 - 2.416) | < 0.001 | 2.050 (1.188 - 3.539) | 0.010 |
| Histologic grade | 524 |  | < 0.001 |  |  |
| G1 | 14 | Reference |  | Reference |  |
| G2 | 228 | 7537225.9701 (0.000 - Inf) | 0.993 | 6365542.7912 (0.000 - Inf) | 0.995 |
| G3 | 206 | 13735528.0667 (0.000 - Inf) | 0.993 | 5847161.9811 (0.000 - Inf) | 0.995 |
| G4 | 76 | 37109580.6512 (0.000 - Inf) | 0.993 | 10615386.5102 (0.000 - Inf) | 0.995 |
| Serum calcium | 364 |  | < 0.001 |  |  |
| Low | 204 | Reference |  | Reference |  |
| Normal | 150 | 1.254 (0.885 - 1.776) | 0.203 | 0.780 (0.445 - 1.367) | 0.385 |
| Elevated | 10 | 4.846 (2.404 - 9.769) | < 0.001 | 0.558 (0.141 - 2.208) | 0.406 |
| Hemoglobin | 452 |  | < 0.001 |  |  |
| Low | 262 | Reference |  | Reference |  |
| Normal | 185 | 0.441 (0.309 - 0.629) | < 0.001 | 0.617 (0.343 - 1.111) | 0.107 |
| Elevated | 5 | 2.645 (0.839 - 8.343) | 0.097 | 1.067 (0.127 - 8.964) | 0.952 |
| Laterality | 531 |  | 0.022 |  |  |
| Left | 250 | Reference |  | Reference |  |
| Right | 281 | 0.707 (0.525 - 0.952) | 0.023 | 1.200 (0.734 - 1.964) | 0.468 |
| ERVFRD-1 | 532 |  | < 0.001 |  |  |
| Low | 266 | Reference |  | Reference |  |
| High | 266 | 0.594 (0.439 - 0.804) | < 0.001 | 0.496 (0.293 - 0.839) | 0.009 |

Abbreviations: KIRC, Kidney Renal Clear Cell Carcinoma; CI, confidence interval.
