# Supplementary material for: Endogenous retrovirus group FRD member 1 is a potential biomarker for prognosis and immunotherapy for kidney renal clear cell carcinoma
Source: Front Cell Infect Microbiol. 2023 Sep 13;13:1252905. doi: 10.3389/fcimb.2023.1252905 (PMC10534008; doi:10.3389/fcimb.2023.1252905)
Supplement: Supplementary file 9 [file Table_6.docx]

Supplementary Table S6

Univariate and multivariate analyses of disease-specific survival in patients with KIRC.

| Characteristics | Total(N) | HR(95% CI) Univariate analysis | P value Univariate analysis | HR(95% CI) Multivariate analysis | P value Multivariate analysis |
| --- | --- | --- | --- | --- | --- |
| Pathologic T stage | 521 |  | < 0.001 |  |  |
| T1 | 270 | Reference |  | Reference |  |
| T2 | 68 | 4.439 (2.187 - 9.008) | < 0.001 | 0.299 (0.021 - 4.271) | 0.373 |
| T3 | 173 | 8.716 (4.906 - 15.485) | < 0.001 | 0.869 (0.088 - 8.537) | 0.904 |
| T4 | 10 | 32.779 (14.154 - 75.913) | < 0.001 | 1.248 (0.071 - 21.808) | 0.879 |
| Pathologic N stage | 254 |  | 0.003 |  |  |
| N0 | 239 | Reference |  | Reference |  |
| N1 | 15 | 3.832 (1.815 - 8.090) | < 0.001 | 0.289 (0.040 - 2.105) | 0.220 |
| Pathologic M stage | 489 |  | < 0.001 |  |  |
| M0 | 414 | Reference |  | Reference |  |
| M1 | 75 | 9.084 (6.202 - 13.307) | < 0.001 | 1.715 (0.112 - 26.206) | 0.698 |
| Pathologic stage | 518 |  | < 0.001 |  |  |
| Stage I | 264 | Reference |  | Reference |  |
| Stage II | 56 | 4.238 (1.682 - 10.678) | 0.002 | 6.599 (0.361 - 120.549) | 0.203 |
| Stage III | 119 | 7.727 (3.657 - 16.324) | < 0.001 | 5.275 (0.435 - 63.993) | 0.192 |
| Stage IV | 79 | 30.952 (15.363 - 62.357) | < 0.001 | 22.158 (0.538 - 911.869) | 0.102 |
| Gender | 521 |  | 0.352 |  |  |
| Female | 181 | Reference |  |  |  |
| Male | 340 | 1.212 (0.805 - 1.823) | 0.358 |  |  |
| Race | 514 |  | 0.431 |  |  |
| Asian&Black or African American | 63 | Reference |  |  |  |
| White | 451 | 1.344 (0.623 - 2.897) | 0.451 |  |  |
| Age | 521 |  | 0.125 |  |  |
| <= 60 | 260 | Reference |  |  |  |
| > 60 | 261 | 1.342 (0.920 - 1.958) | 0.126 |  |  |
| Histologic grade | 513 |  | < 0.001 |  |  |
| G1 | 14 | Reference |  | Reference |  |
| G2 | 227 | 3107837.6909 (0.000 - Inf) | 0.994 | 2169661.3662 (0.000 - Inf) | 0.997 |
| G3 | 198 | 9271092.3878 (0.000 - Inf) | 0.994 | 1511764.1753 (0.000 - Inf) | 0.997 |
| G4 | 74 | 30850439.5481 (0.000 - Inf) | 0.993 | 3764780.1982 (0.000 - Inf) | 0.997 |
| Serum calcium | 358 |  | 0.001 |  |  |
| Low | 200 | Reference |  | Reference |  |
| Normal | 148 | 1.364 (0.880 - 2.115) | 0.165 | 0.550 (0.264 - 1.146) | 0.110 |
| Elevated | 10 | 5.994 (2.671 - 13.450) | < 0.001 | 0.687 (0.181 - 2.615) | 0.582 |
| Hemoglobin | 444 |  | < 0.001 |  |  |
| Low | 256 | Reference |  | Reference |  |
| Normal | 183 | 0.433 (0.277 - 0.677) | < 0.001 | 1.043 (0.482 - 2.256) | 0.914 |
| Elevated | 5 | 2.667 (0.653 - 10.884) | 0.172 | 0.000 (0.000 - Inf) | 0.998 |
| Laterality | 520 |  | 0.015 |  |  |
| Left | 246 | Reference |  | Reference |  |
| Right | 274 | 0.627 (0.429 - 0.918) | 0.016 | 1.096 (0.593 - 2.028) | 0.769 |
| ERVFRD-1 | 521 |  | < 0.001 |  |  |
| Low | 261 | Reference |  | Reference |  |
| High | 260 | 0.373 (0.247 - 0.563) | < 0.001 | 0.375 (0.183 - 0.770) | 0.008 |

Abbreviations: KIRC, Kidney Renal Clear Cell Carcinoma; CI, confidence interval.
