# Supplementary material for: Endogenous retrovirus group FRD member 1 is a potential biomarker for prognosis and immunotherapy for kidney renal clear cell carcinoma
Source: Front Cell Infect Microbiol. 2023 Sep 13;13:1252905. doi: 10.3389/fcimb.2023.1252905 (PMC10534008; doi:10.3389/fcimb.2023.1252905)
Supplement: Supplementary file 10 [file Table_7.docx]

Supplementary Table S7

Univariate and multivariate analyses of progress free interval in patients with KIRC.

| Characteristics | Total(N) | HR(95% CI) Univariate analysis | P value Univariate analysis | HR(95% CI) Multivariate analysis | P value Multivariate analysis |
| --- | --- | --- | --- | --- | --- |
| Pathologic T stage | 530 |  | < 0.001 |  |  |
| T1 | 272 | Reference |  | Reference |  |
| T2 | 69 | 3.460 (2.054 - 5.830) | < 0.001 | 4.500 (0.397 - 51.057) | 0.225 |
| T3 | 178 | 6.289 (4.183 - 9.457) | < 0.001 | 8.381 (0.862 - 81.453) | 0.067 |
| T4 | 11 | 17.536 (8.557 - 35.935) | < 0.001 | 1.800 (0.142 - 22.835) | 0.650 |
| Pathologic N stage | 255 |  | 0.001 |  |  |
| N0 | 239 | Reference |  | Reference |  |
| N1 | 16 | 3.663 (1.882 - 7.131) | < 0.001 | 0.601 (0.154 - 2.349) | 0.465 |
| Pathologic M stage | 498 |  | < 0.001 |  |  |
| M0 | 421 | Reference |  | Reference |  |
| M1 | 77 | 8.922 (6.438 - 12.363) | < 0.001 | 0.794 (0.071 - 8.816) | 0.851 |
| Pathologic stage | 527 |  | < 0.001 |  |  |
| Stage I | 266 | Reference |  | Reference |  |
| Stage II | 57 | 2.457 (1.282 - 4.709) | 0.007 | 0.297 (0.022 - 4.021) | 0.361 |
| Stage III | 123 | 4.735 (2.931 - 7.652) | < 0.001 | 0.555 (0.053 - 5.862) | 0.624 |
| Stage IV | 81 | 20.135 (12.771 - 31.746) | < 0.001 | 16.523 (0.538 - 507.398) | 0.108 |
| Gender | 530 |  | 0.019 |  |  |
| Female | 186 | Reference |  | Reference |  |
| Male | 344 | 1.502 (1.060 - 2.126) | 0.022 | 1.999 (1.095 - 3.646) | 0.024 |
| Race | 523 |  | 0.340 |  |  |
| Asian&Black or African American | 64 | Reference |  |  |  |
| White | 459 | 1.319 (0.730 - 2.382) | 0.359 |  |  |
| Age | 530 |  | 0.101 |  |  |
| <= 60 | 263 | Reference |  |  |  |
| > 60 | 267 | 1.298 (0.950 - 1.773) | 0.102 |  |  |
| Histologic grade | 522 |  | < 0.001 |  |  |
| G1 | 14 | Reference |  | Reference |  |
| G2 | 228 | 1.524 (0.208 - 11.137) | 0.678 | 0.061 (0.007 - 0.561) | 0.013 |
| G3 | 206 | 3.742 (0.520 - 26.946) | 0.190 | 0.052 (0.005 - 0.493) | 0.010 |
| G4 | 74 | 13.282 (1.834 - 96.189) | 0.010 | 0.153 (0.015 - 1.518) | 0.109 |
| Serum calcium | 363 |  | 0.005 |  |  |
| Low | 203 | Reference |  | Reference |  |
| Normal | 150 | 1.317 (0.909 - 1.909) | 0.145 | 0.335 (0.167 - 0.671) | 0.002 |
| Elevated | 10 | 4.533 (2.061 - 9.971) | < 0.001 | 0.319 (0.090 - 1.134) | 0.077 |
| Hemoglobin | 450 |  | 0.009 |  |  |
| Low | 261 | Reference |  | Reference |  |
| Normal | 184 | 0.598 (0.421 - 0.849) | 0.004 | 1.865 (0.984 - 3.532) | 0.056 |
| Elevated | 5 | 1.672 (0.412 - 6.789) | 0.472 | 0.000 (0.000 - Inf) | 0.997 |
| Laterality | 529 |  | 0.007 |  |  |
| Left | 248 | Reference |  | Reference |  |
| Right | 281 | 0.652 (0.477 - 0.892) | 0.007 | 0.729 (0.425 - 1.248) | 0.249 |
| ERVFRD-1 | 530 |  | < 0.001 |  |  |
| Low | 265 | Reference |  | Reference |  |
| High | 265 | 0.484 (0.349 - 0.671) | < 0.001 | 0.708 (0.387 - 1.297) | 0.264 |

Abbreviations: KIRC, Kidney Renal Clear Cell Carcinoma; CI, confidence interval.
